# Influence of Treadmill Speed and Perturbation Intensity on Selection of Balancing Strategies during Slow Walking Perturbed in the Frontal Plane

**DOI:** 10.1155/2019/1046459

**Published:** 2019-06-02

**Authors:** Zlatko Matjačić, Matjaž Zadravec, Andrej Olenšek

**Affiliations:** University Rehabilitation Institute, Republic of Slovenia, Linhartova 51, SI-1000 Ljubljana, Slovenia

## Abstract

**Background:**

Common understanding is that adequate foot placement (stepping strategy) is crucial in maintaining stability during walking at normal speed. The aim of this study was to investigate strategies that humans use to cope with lateral perturbations during very slow walking.

**Methods:**

Ten healthy individuals underwent an experimental protocol whereby a set of perturbations directed inward (medially to a stance leg) and outward (laterally to a stance leg) of three intensities (*F*_1_ = 5%, *F*_2_ = 10%, and *F*_3_ = 15% of body weight), applied at three instances of a stance phase, were delivered in random order to the pelvis using a balance assessment robot while walking on a treadmill at three walking speeds (*S*_1_ = 0.4, *S*_2_ = 0.6, and *S*_3_ = 0.8 m/s). We analyzed the peak center of mass displacements; step length, step width, and step times; and the lateral component of ground reaction force for perturbations that were delivered at the beginning of the gait cycle.

**Results:**

Responses after inward perturbations were similar at all tested speeds and consistently employed stepping strategy that was further facilitated by a shortened stance. Wider and shorter steps were applied with increased perturbation intensity. Responses following outward perturbations were more complex. At *S*_1_, hip strategy (impulse-like increase of mediolateral ground reaction force) augmented with ankle strategy (mediolateral shift of the center of pressure) mainly contributed to responses already during the stance phase. The stance duration was significantly longer for all perturbation intensities. At *S*_2_, the relative share of hip strategy was reduced while with increased perturbation intensity, stepping strategy was gradually added. The stance duration was significantly longer for *F*_1_ and *F*_2_. At *S*_3_, stepping strategy was mainly used while the duration of stance was similar to the one in unperturbed walking. Responses following both inward and outward perturbations at all speeds were characterized by temporary slowing down movement in a sagittal plane that was more pronounced with increased perturbation intensity.

**Conclusions:**

This study provides novel insights into balancing strategies used at slower walking speeds which may be more relevant to understand the challenges of gait stability following perturbations in the frontal plane in clinical populations.

## 1. Introduction

An essential component of bipedal walking is maintenance of dynamic balance, particularly in the frontal plane [1]. The main mechanism used in normal unperturbed human walking has been explained through the inverted pendulum model and is related to adequate placement of the swinging limb onto a new stance location [2]. This changes the base of support (BOS) and provides appropriate development of the lateral component of ground reaction force to ensure stable side-to-side movement of the center of mass (COM) [3].

Likewise, following a perturbation that may be imposed by various perturbation modalities, for example, (i) as a push at the waist level, mimicking a sudden bump into another person in a crowd [4–7], (ii) as a movement of the support surface, mimicking a slip [8], and (iii) as a pull on the foot of the swinging leg, mimicking a trip [9], the main balancing strategy used was related to the placement of the swinging limb onto an adequate location [3, 10, 11]. Stepping was additionally augmented by the “ankle strategy,” which is related to the activity of ankle musculature to displace the center of pressure (COP) under the stance leg in the direction of the action of perturbation [12, 13]. Additionally, for perturbations acting in the inward direction (medially relative to the stance leg in case of perturbing pushes to the waist), the swing time was shortened to facilitate earlier application of balance correction in the next step [4, 6]. However, for the perturbations acting in the outward direction (laterally relative to the stance leg in case of perturbing pushes to the waist), shortening of the swing phase has not been observed [4, 6]. Several studies have also pointed out that in sessions where perturbations in the frontal plane were delivered subjects adopted wider stepping as an additional stabilizing measure, compared to sessions without perturbations [14, 15]. The above referenced studies examined dynamic reactions to perturbations that were imposed during walking in a range of speeds that are normally used (0.8–1.2 m/s).

Various diseases or injuries to the central nervous system (CNS) result in substantially reduced motor capabilities in clinical cases. For example, after completion of clinical rehabilitation, the majority of stroke survivors walk with speeds that range from 0.4 to 0.8 m/s [16]. Our knowledge of balancing mechanisms used following perturbations at these lower speeds of walking is scarce. One consequence of slower walking is that swing times are longer. Thus, for example, if a perturbation is imposed during a double support phase, which would resemble a situation of a slip on the floor [8], it may be the case that a corrective action coming from a wider/narrower next step, which inevitably acts with considerable delay against the induced instability [4], would not be sufficient to successfully correct for the perturbation. Thus, corrective actions may be required to start already during the stance phase. Apart from using ankle strategy under the stance leg that can act fast against perturbation but has limited stabilizing effect due to a narrow foot width [4, 13], additional strategy related to counter-rotation of body segments, termed as “inertial strategy” [12], which is frequently used during one-leg standing [17], may be utilized during slow walking. The most notable example of inertial strategy is related to the pelvis and trunk rotation and has been termed as “hip strategy” [12, 17]. In our previous work with a selected neurologically intact subject walking at the speed of 0.4 m/s, we observed that an important contribution to the balancing response after an outward perturbing push was a hip strategy related to the activity of hip abductors of the stance leg [18]. Vlutters et al. [19] have also observed important activity of the gluteus medius muscle of the stance leg following outward pushes at walking speed of 0.6 m/s. On the other hand, studies from Hof et al. [4] and Vlutters et al. [19] where pelvis perturbations of similar intensity were applied in the frontal plane at walking speed of 1.2 m/s have not observed use of hip strategy. This indicates that walking speed may have a considerable influence on the selection of a suitable balancing strategy or a synergy of balancing strategies following perturbations applied in the frontal plane.

The aim of this study was to systematically investigate the kinematics and kinetics of reactive dynamic balancing at various speeds of slower walking and at various intensities of inward- and outward-directed perturbing pushes applied at the waist at the beginning of the stance phase, to elucidate the interplay of strategies that humans use to cope with the consequences of an unexpected lateral perturbation.

## 2. Methods

### 2.1. Subjects

Ten healthy males without known history of neuromuscular or orthopedic problems (age: 31 ± 5 years, height: 180 ± 3.9 cm, and mass: 78.7 ± 6.5 kg) participated in this study after signing informed consent forms. The subjects represent a sample of convenience. The study was approved by the Slovenian National Ethics Committee.

### 2.2. Instrumentation

**Figure 1:**
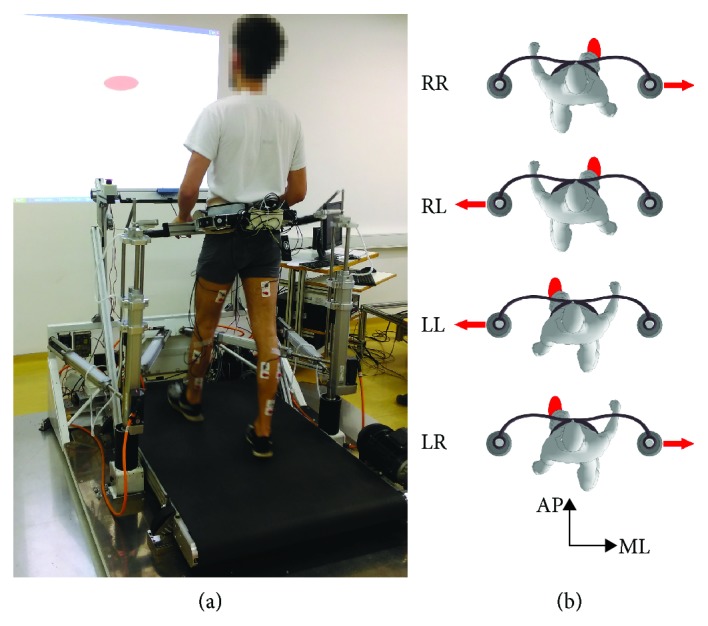
Photo of a subject walking on an instrumented treadmill while being embraced by the BAR-TM perturbing device; projection on the wall shows the middle of the BAR-TM working space as well as the current position and orientation of the pelvis in a transverse plane—the subjects were instructed to return to the middle of the BAR-TM working space after they rejected perturbation (a). Top view illustration of perturbation directions: outward RR: perturbation to the right triggered at right-foot contact; inward RL: perturbation to the left triggered at right-foot contact; outward LL: perturbation to the left triggered at left-foot contact; inward LR: perturbation to the right triggered at left-foot contact (b).

Figure 1 shows the experimental environment, which consisted of a balance assessment robot and an instrumented treadmill (BART). Here, only a brief description of the experimental setup is given, as a more detailed description is provided elsewhere [6, 7]. The BART interfaces with the pelvis of a walking participant with six degrees of freedom (DOF). Five of the DOFs (translation of the pelvis in the sagittal, lateral, and vertical directions; pelvic rotation; and pelvic list) are actuated and admittance-controlled, providing transparent haptic interaction with negligible power transfer [7]. The sixth DOF (pelvic tilt) is passive. The BAR-TM is capable of delivering perturbations in the forward/backward and left/right directions. In this study, we only considered inward and outward perturbations delivered in the frontal plane as depicted in Figure 1.

COM movement was estimated from the translational movement of the subjects' pelvis and assessed from the movement of the BAR-TM, similarly as in our previous studies [7, 18]. Recordings of the ground reaction force (GRF) and COP in the transversal plane during walking were obtained by means of four force transducers (K3D120, ME Systeme GmbH) placed underneath the treadmill. Spatiotemporal data were assessed by means of an OptiTrack camera (NaturalPoint Inc.). Passive reflective markers were placed on the participants' feet (on the medial malleoli and the first and fourth metatarsal joints) [7, 18]. Sampling frequency for the kinematic and kinetic data was 50 Hz which is considered to be adequate for this type of study [20].

### 2.3. Experimental Protocol

**Figure 2:**
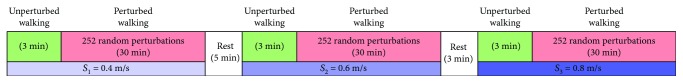
Schematic diagram of the experimental protocol.

The experimental protocol is shown schematically in Figure 2. First, subjects walked at a treadmill speed set to 0.4 m/s for a period of three minutes—unperturbed walking session. This was followed by a period of around half an hour of perturbed walking—perturbed walking session. These two experimental blocks were then repeated for treadmill speeds of 0.6 m/s and 0.8 m/s. The whole protocol was done in a single day and took around 2 hours. Perturbations were delivered with a randomly varied pause that ranged from six to eight seconds in order to avoid predictability of the perturbation occurrence. Four perturbation directions (outward RR and LL and inward RL and LR), three perturbation onsets (at 0%, 30%, and 60% of the stance phase of a gait cycle), and three perturbation amplitudes (5%, 10%, and 15% of body weight) were varied. Each combination of perturbation parameters was repeated seven times. This yielded a total of 252 perturbing pushes at each walking speed that were block-randomized. Perturbations took the form of a force impulse lasting 150 ms [6, 7, 18]. Prior to this study, all subjects visited our laboratory where they practiced unperturbed and perturbed walking on the BAR-TM system for approximately half an hour.

### 2.4. Measurements and Data Analysis

The COM, COP, and GRF were first segmented into strides with the gait cycle defined as the period between two consecutive left (for LL and LR responses) or right (for RR and RL responses) heel strikes, as detected from COP_ML_ and COP_AP_ signals. Two full gait cycles, half of a cycle prior to and one and a half cycles after the onset of perturbation, were analyzed. Spatiotemporal responses were investigated in terms of step length, step width, and step time where left (right) step length was taken to be the anterio-posterior distance between ankle markers at the moment of left (right) foot strike while left (right) step width was defined as the mediolateral distance between the same markers. Step times were defined as the time elapsed between two consecutive left (right) and right (left) foot strikes. In each combination of perturbation parameters, COM, COP, and GRF trajectories and spatiotemporal parameters were averaged across seven repetitions. We also averaged spatiotemporal parameters for unperturbed walking in unperturbed walking sessions and unperturbed walking (the periods between the complete recoveries from previous perturbation until the onset of the next perturbation) in the perturbed walking sessions at each tested treadmill speed.

Although we assessed postural responses at three levels of perturbation onset, we included in further analysis only perturbations that commenced at 0% of a gait cycle.

The following data were used as outcome measures: step lengths, step widths, and step times for perturbed (we analyzed the first step after the perturbation onset which determines the “stepping” response) and unperturbed experimental conditions; peak displacements of COM within the first stride (from 0% to 100% of the gait cycle) in sagittal (COM_AP peak_) and frontal planes (COM_ML peak_); and integral of the lateral component of GRF (GRF_ML impulse_) for the period of the first stance phase (from 0% to approx. 50% of a gait cycle) (“in-stance response”) and for the period of the second stance phase (from approx. 50% to 100% of a gait cycle) (“stepping response”). Thus, the “in-stance response” period encompassed the balancing activity prior to the first step after the onset of perturbation, while the “stepping response” period encompassed the balancing activity between the first and the second steps after the onset of perturbation. Since GRF_ML_ determines the acceleration of COM_ML_, the GRF_ML impulse_ provides a measure of the overall balancing activity in both “in-stance” and “stepping” periods of balance responses.

### 2.5. Statistical Analysis

For unperturbed walking, a two-way repeated measures analysis of variance (rmANOVA) was used to test for the main effects and interactions on step length, step width, and step time between walking speed (3 levels: 0.4, 0.6, and 0.8 m/s) and walking condition (2 levels: unperturbed walking during unperturbed walking sessions and unperturbed walking during perturbed walking sessions). When a significant main effect or interaction was found, we performed post hoc pairwise comparisons for each of the walking speeds separately. A significance level of 0.05 was used.

For perturbed walking, a two-way rmANOVA was used to test for the main effects and interactions on step length, step width, step time, COM_ML peak_, COM_AP peak_, and GRF_ML impulse_ between walking speed (3 levels: 0.4, 0.6, and 0.8 m/s) and perturbation amplitude (4 levels: 0% (unperturbed strides from perturbed sessions), 5%, 10%, and 15% of body weight). When a significant main effect or interaction was found, we performed post hoc pairwise comparisons versus unperturbed walking for each of the walking speeds separately. A significance level of 0.05 was used, and a Bonferroni correction was applied to correct for multiple comparisons (0.016).

## 3. Results

The results for pushes RR (outward perturbation) and RL (inward perturbation) are presented in this section. The effects of pushes to both outward directions (LL and RR) were comparable. Likewise, the effects of pushes to both inward directions (LR and RL) were comparable.

### 3.1. Dynamic Balancing Responses following Perturbations

#### 3.1.1. Outward Perturbations

**Figure 3:**
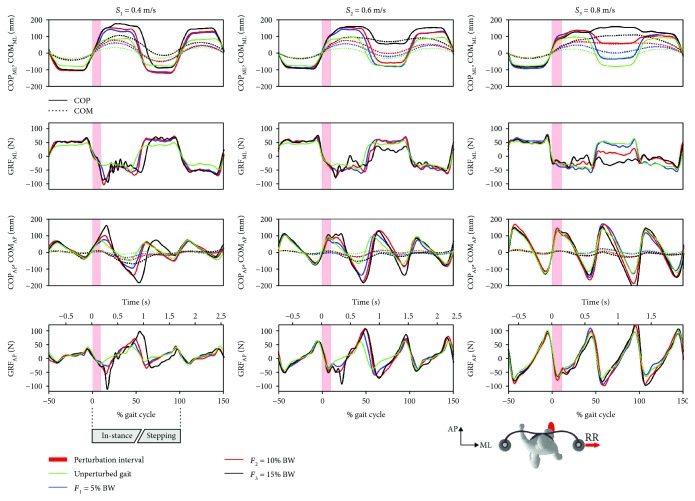
Kinematics and kinetics of balancing responses following outward RR perturbation assessed in a representative subject. The first row shows the trajectories of COP_ML_ (solid lines) and COM_ML_ (dotted lines), while the second row shows GRF_ML_ trajectories. The third row shows COP_AP_ (solid lines) and COM_AP_ (dotted lines) trajectories, while the fourth row shows GRF_AP_ trajectories. Each graph contains responses to all three perturbation intensities along with the trajectories assessed during the unperturbed walking sessions. The left, middle, and right columns show the balancing responses at speeds *S*_1_, *S*_2_, and *S*_3_, respectively. Half a stride prior to and one and a half strides following the perturbation commencement are shown. A stride is defined as the period between two consecutive right-foot contacts. The trajectories displayed show mean values of seven balancing responses.

Figure 3 shows COP, COM, and GRF responses to outward perturbations (RR) for all three tested walking speeds and for all three tested perturbation intensities for a representative subject.

*(1) Frontal Plane*. At a walking speed of 0.4 m/s, we can observe increased lateral displacement of COP_ML_ in the “in-stance” period of the response (from 0% to approx. 50% of a gait cycle) in relation to unperturbed walking. An impulse-like rise in the GRF_ML_ can be seen in the first half of the stance that is similar for all three intensities and acts in the direction opposite to the perturbation. Perturbation was fully contained during the “in-stance” period for perturbation intensities of 5% and 10% while following a perturbation intensity of 15%, there was medial displacement of COP_ML_ and related decrease in GRF_ML_ in the “stepping period” (from approx. 50% to approx. 100% of a gait cycle) that finally contained the instability.

At a walking speed of 0.6 m/s, we can observe increased lateral displacement of COP_ML_ in the “in-stance” period while the impulse-like rise in GRF_ML_ in the first half of the “in-stance” period was smaller in comparison to those at walking speed 0.4 m/s. Medial displacement of COP_ML_ and related decrease in GRF_ML_ were observed in the “stepping” period for the perturbation intensity of 15%.

At a walking speed of 0.8 m/s, increased lateral displacement of COP_ML_ in the “in-stance” period was observed while the impulse-like rise in the GRF_ML_ in the first half of the “in-stance” period was not present. In the second half of the same period, there was a gradual decrease of GRF_ML_ with increasing perturbation intensity followed by a progressively larger medial displacement of COP_ML_ and related decrease in GRF_ML_ in the “stepping” period.

*(2) Sagittal Plane*. At walking speed of 0.4 m/s, COP_AP_ was displaced increasingly forward in the first half of the stance with increasing intensity of perturbation while GRF_AP_ showed increased braking action that decelerated COM_AP_ in relation to unperturbed walking. Slowing down of COM_AP_ and associated changes in COP_AP_ were progressively smaller at walking speeds of 0.6 m/s and 0.8 m/s compared to those observed at the speed of 0.4 m/s.

#### 3.1.2. Inward Perturbations

**Figure 4:**
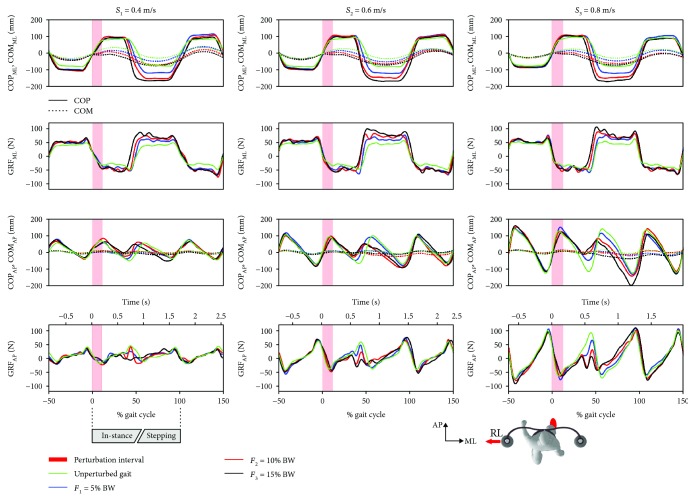
Kinematics and kinetics of balancing responses following inward RL perturbation assessed in a representative subject. The first row shows the trajectories of COP_ML_ (solid lines) and COM_ML_ (dotted lines), while the second row shows GRF_ML_ trajectories. The third row shows COP_AP_ (solid lines) and COM_AP_ (dotted lines) trajectories, while the fourth row shows GRF_AP_ trajectories. Each graph contains responses to all three perturbation intensities along with the trajectories assessed during the unperturbed walking sessions. The left, middle, and right columns show the balancing responses at speeds *S*_1_, *S*_2_, and *S*_3_, respectively. Half a stride prior to and one and a half strides following the perturbation commencement are shown. A stride is defined as the period between two consecutive right-foot contacts. The trajectories displayed show mean values of seven balancing responses.

Figure 4 shows COP, COM, and GRF responses to inward perturbations (RL) for all three tested walking speeds and for all three tested perturbation intensities for a representative subject. The responses look similar across the tested walking speeds.

*(1) Frontal Plane*. In the “in-stance” period, no noticeable difference can be observed in COP_ML_, COM_ML_, and GRF_ML_ in relation to unperturbed walking except for a shortened duration of the stance phase. The dominant balancing response can be observed in the “stepping” period where depending on the perturbation intensity COP_ML_ was shifted laterally which was accompanied with a progressively increased GRF_ML_.

*(2) Sagittal Plane*. In the second part of the “in-stance” period, a shortened posterior displacement of COP_AP_ can be observed. Consequently, GRF_AP_ was also reduced thus slowing down movement of COM_AP_. Throughout the “stepping” period, a smaller anterior displacement of COP_AP_ can be seen with accompanying reduction of GRF_AP_ which enabled COM_AP_ to catch up with the relative position of COM_AP_ on the treadmill that the subject assumed before the action of perturbation.

### 3.2. Peak COM Displacements

**Figure 5:**
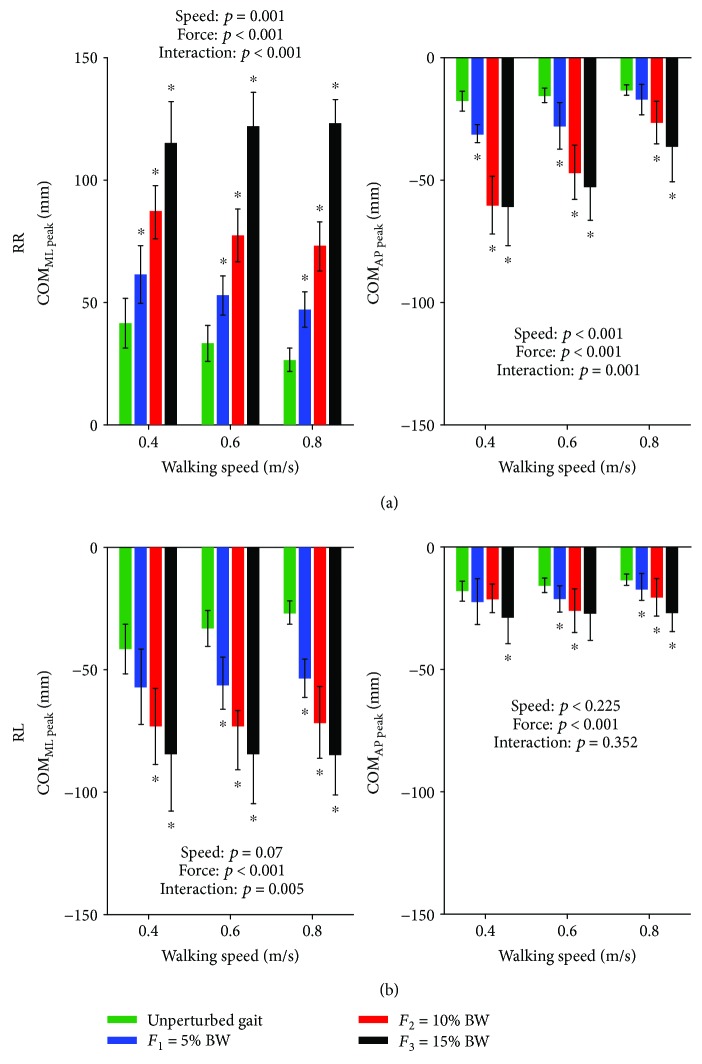
Group average (±standard deviation) of peak COM_ML_ and COM_AP_ excursions across the three walking speeds during unperturbed walking and perturbed walking is shown for outward RR (a) and inward RL (b) perturbations along with the *p* values of 2-way rmANOVA. Asterisks (^∗^) indicate significant difference from unperturbed walking in Bonferroni post hoc pairwise comparisons (*p* < 0.016).

Figure 5 shows peak excursions of COM_ML_ and COM_AP_ for both outward (RR) and inward (RL) perturbations. COM_ML peak_ following outward perturbation was significantly affected by the speed of walking (*F*(2, 18) = 10.015, *p* = 0.001), the perturbation intensity (*F*(3, 27) = 274.194, *p* < 0.001), and their interaction (*F*(6, 54) = 9.790, *p* < 0.001). Post hoc analysis has shown significantly larger peak COM_ML_ displacements for all intensities and at all speeds in comparison to unperturbed walking. COM_AP peak_ following outward perturbation was significantly affected by the speed of walking (*F*(2, 18) = 69.523, *p* < 0.001), the perturbation intensity (*F*(3, 27) = 88.255, *p* < 0.001), and their interaction (*F*(6, 54) = 5.551, *p* < 0.001). Post hoc analysis has shown significantly larger peak COM_AP_ displacements for all intensities and at all speeds in comparison to unperturbed walking. COM_ML peak_ following inward perturbation was significantly affected by the intensity of perturbation (*F*(3, 27) = 53.150, *p* < 0.001) and interaction between intensity and speed (*F*(6, 54) = 3.556, *p* = 0.005) but not by the speed of walking (*F*(2, 18) = 3.088, *p* = 0.070). Post hoc analysis has shown significantly larger peak COM_ML_ displacements for all intensities and at all speeds in comparison to unperturbed walking. COM_AP peak_ following inward perturbation was significantly affected only by the perturbation intensity (*F*(3, 27) = 18.892, *p* < 0.001) but not by the walking speed (*F*(2, 18) = 1.620, *p* = 0.225) nor the interaction between the intensity and walking speed (*F*(6, 54) = 1.140, *p* = 0.352). Post hoc analysis has shown significantly larger peak COM_ML_ displacements for majority of intensities at all speeds in comparison to unperturbed walking.

### 3.3. Spatiotemporal Parameters

**Figure 6:**
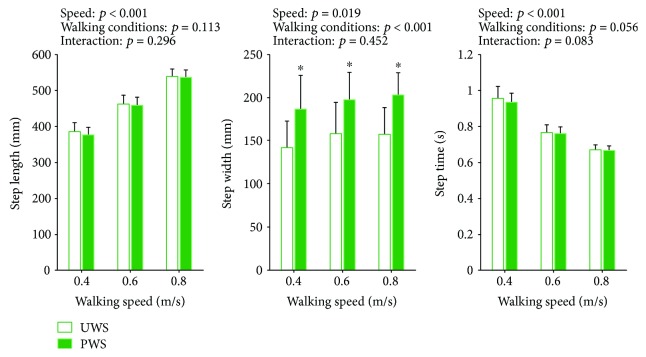
Group average (±standard deviation) of step lengths, step widths, and step times across the three walking speeds during unperturbed walking in unperturbed walking session (UWS) and during unperturbed walking in perturbed walking sessions (PWS) is shown along with the *p* values of 2-way rmANOVA. Asterisks (^∗^) indicate significant difference from unperturbed walking in post hoc pairwise comparisons (*p* < 0.05).

Figure 6 shows spatiotemporal parameters for unperturbed walking during the unperturbed walking sessions (UWS) and for unperturbed walking during the perturbed walking sessions (PWS). Step lengths and step times were significantly affected only by the walking speed (step length *F*(2, 18) = 354.221, *p* < 0.001; step time *F*(2, 18) = 238.195, *p* < 0.001) but not by the walking condition (step length *F*(1, 9) = 3.089, *p* = 0.113; step time *F*(1, 9) = 4.822, *p* = 0.056) nor the interaction between the walking condition and the walking speed (step length *F*(2, 18) = 1.304, *p* = 0.296; step time *F*(2, 18) = 2.866, *p* = 0.083). Step lengths increased with increased walking speed while the step times decreased with increased walking speed. Step widths were significantly affected by walking speed (*F*(2, 18) = 4.996, *p* = 0.019) and walking condition (*F*(1, 9) = 70.489, *p* < 0.001) but not by interaction of the two (*F*(2, 18) = 0.830, *p* = 0.452). Post hoc analysis has shown that the difference between step widths of unperturbed walking during unperturbed walking sessions and during perturbed walking sessions was on average 4 cm.

**Figure 7:**
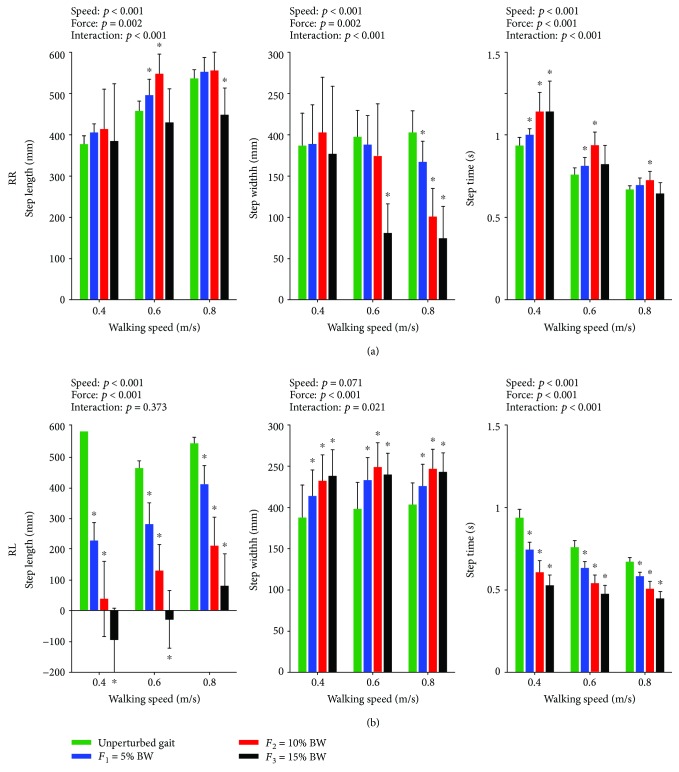
Group average (±standard deviation) of step lengths, step widths, and step times across the three walking speeds during unperturbed walking and perturbed walking is shown for outward RR (a) and inward RL (b) perturbations along with the *p* values of 2-way rmANOVA. Asterisks (^∗^) indicate significant difference from unperturbed walking in Bonferroni post hoc pairwise comparisons (*p* < 0.016).

Figure 7 shows spatiotemporal parameters for perturbed walking following outward (RR) and inward (RL) perturbations. Step lengths, step widths, and step times following outward perturbation were significantly affected by the walking speed (step length *F*(2, 18) = 38.259, *p* < 0.001; step width *F*(2, 18) = 8.869, *p* = 0.002; and step time *F*(2, 18) = 153.168, *p* < 0.001), by intensity (step length *F*(3, 27) = 6.721, *p* = 0.002; step width *F*(3, 27) = 51.945, *p* < 0.001; and step time *F*(3, 27) = 12.214, *p* < 0.001), and by interaction of both factors (step length *F*(6, 54) = 5.407, *p* < 0.001; step width *F*(6, 54) = 12.023, *p* < 0.001; and step time *F*(6, 54) = 13.333, *p* < 0.001). Post hoc analysis showed no significant differences in step lengths at 0.4 m/s; at 0.6 m/s, significantly longer steps were taken at perturbation intensities of 5% and 10% while at 0.8 m/s, significantly shorter steps were made at a perturbation intensity of 15% in comparison to unperturbed walking. Post hoc analysis further revealed that step widths at a walking speed of 0.4 m/s were not statistically different; at the walking speed of 0.6 m/s, step width at the strongest perturbation was significantly smaller in comparison to unperturbed walking while at a walking speed of 0.8 m/s, step widths for all intensities were significantly smaller in comparison to unperturbed walking. Post hoc analysis for step times has shown significantly longer steps at 0.4 m/s for all intensities in comparison to unperturbed walking; at 0.6 m/s, this was the case for intensities of 5% and 10% while at 0.8 m/s, the step time for an intensity of 10% was significantly longer in comparison to unperturbed walking.

Step lengths and step times following inward perturbation were significantly affected by the walking speed (step length *F*(2, 18) = 43.703, *p* < 0.001; step time *F*(2, 18) = 75.724, *p* < 0.001) but not the step width (*F*(2, 18) = 3.085, *p* = 0.071). On the other hand, perturbation intensity had significant effect on step length, step width, and step time (step length *F*(3, 27) = 195.329, *p* < 0.001; step width *F*(3, 27) = 31.060, *p* < 0.001; and step time *F*(3, 27) = 361.699, *p* < 0.001). Only step width and step time were significantly affected by interaction of both factors (step width *F*(6, 54) = 2.758, *p* = 0.021; step time *F*(6, 54) = 44.580, *p* < 0.001) and not the step length (*F*(6, 54) = 1.103, *p* = 0.373). Post hoc analysis has shown a decrease of step lengths and step times and an increase of step widths with increased intensity at all tested speeds in comparison to unperturbed walking.

### 3.4. Lateral GRF Impulses

**Figure 8:**
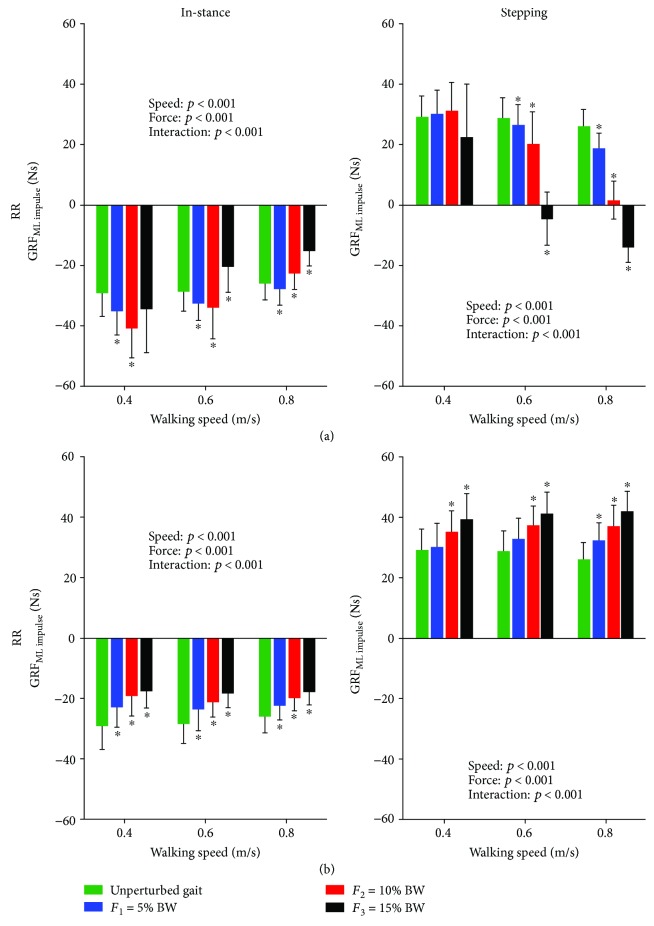
Group average (±standard deviation) of integrals of GRF_ML_ over the “in-stance” and “stepping” periods across the three walking speeds during unperturbed walking and perturbed walking is shown for outward RR (a) and inward RL (b) perturbations along with the *p* values of 2-way rmANOVA. Asterisks (^∗^) indicate significant difference from unperturbed walking in Bonferroni post hoc pairwise comparisons (*p* < 0.016).

Figure 8 shows the integral of GRF_ML_ over the “in-stance” and “stepping” periods of dynamic responses following outward (RR) and inward (RL) perturbations. GRF_ML impulse_ following outward perturbation was significantly affected by the walking speed (“in-stance” *F*(2, 18) = 37.079, *p* < 0.001; “stepping” *F*(2, 18) = 82.463, *p* < 0.001), by intensity (“in-stance” *F*(3, 27) = 26.849, *p* < 0.001; “stepping” *F*(3, 27) = 87.569, *p* < 0.001), and by interaction of both factors (“in-stance” *F*(6, 54) = 10.037, *p* < 0.001; “stepping” *F*(6, 54) = 24.318, *p* < 0.001) during both periods. Post hoc analysis for the “in-stance” period has shown significant increases for intensities of 5% and 10% at a walking speed of 0.4 m/s while GRF_ML impulse_ value at an intensity of 15% was not significantly different from unperturbed walking. At 0.6 m/s, GRF_ML impulse_ values at intensities of 5% and 10% were significantly increased while GRF_ML impulse_ value at an intensity of 15% was significantly decreased in comparison to unperturbed walking. At 0.8 m/s, only GRF_ML impulse_ value at an intensity of 5% was significantly increased while GRF_ML impulse_ values at intensities of 10% and 15% were significantly decreased. Post hoc analysis for the “stepping” period has shown no statistically significant differences among intensity factors at a walking speed of 0.4 m/s. At walking speeds 0.6 m/s and 0.8 m/s, the GRF_ML impulse_ shows increasingly smaller values with increasing intensity of perturbation in comparison to unperturbed walking.

Following inward perturbation, GRF_ML impulse_ was significantly affected in both periods by the perturbation intensity (“in-stance” *F*(3, 27) = 53.116, *p* < 0.001; “stepping” *F*(3, 27) = 28.598, *p* < 0.001) and by interaction of both factors (“in-stance” *F*(6, 54) = 4.832, *p* = 0.001; “stepping” *F*(6, 54) = 7.391, *p* < 0.001) but was not significantly affected by walking speed (“in-stance” *F*(2, 18) = 1.289, *p* = 0.300; “stepping” *F*(2, 18) = 1.319, *p* = 0.292). Post hoc analysis for the “in-stance” period at all speeds showed increasingly smaller values of GRF_ML impulse_ with increasing intensity of perturbation in comparison to unperturbed walking. Post hoc analysis for the “stepping” period at speeds of 0.4 m/s and 0.6 m/s showed significantly higher values of GRF_ML impulse_ for intensities of 10% and 15% while at 0.8 m/s, significantly higher values were observed for all intensities in comparison to unperturbed walking.

## 4. Discussion

The main aim of this study was to investigate how slower walking speeds and various intensities of perturbing pushes delivered at the heel strike influence the selection of dynamic balancing responses.

Responses after inward perturbations were similar at all tested speeds and consistently employed a predominantly stepping strategy facilitated by a shortened stance. Wider steps and shorter stances were applied with increasing perturbation strengths. The role of hip/inertial balancing strategies was not observed. These observations are well in line with the observations of other studies which were mostly performed at higher walking speeds [4, 5, 7, 11].

On the contrary, when subjects were faced with outward perturbations, additional inertial balancing strategies were used. The predominant inertial strategy associated with the counter-rotation of body segments changing GRF is the hip strategy [12, 17, 21]. Depending on the walking speed and perturbation intensity, the relative contribution of individual balancing strategy also varied. At the slowest walking speed, we consistently observed a significant contribution of hip strategy in the first half of the “in-stance” period. The hip strategy was augmented with an ankle strategy, displacing the COP in the lateral direction away from the direction of perturbation [13]. The stance duration was significantly longer for all perturbation intensities. At medium walking speed, the hip strategy augmented with an ankle strategy was sufficient at the weakest perturbation magnitude while a stepping strategy was gradually added with increasing perturbation intensity. At the highest walking speed, stepping was the main strategy used to counteract the effects of perturbation while the duration of stance was similar to those in unperturbed walking.

### 4.1. Synergy of Balancing Strategies following Outward Perturbations

This study provides an important insight into the balancing strategies used at walking speeds that are well below those normally used and which may be more relevant for understanding the challenges of gait stability following perturbations in the frontal plane in clinical populations. This study complements the existing body of knowledge on the organization of balancing responses during walking following perturbations acting in the frontal plane. The results of this study to some extent challenge the currently accepted opinion that control of human gait is predominantly achieved through foot placement [3–5, 7, 10]. Stepping strategy may well be the primary and energetically wise optimal coping option following perturbations at normal speeds of walking; however, the inherent time-delay associated with swing time needed for a stepping strategy to start acting against a perturbation after an outward perturbation is reciprocal to the walking speed. This becomes critical at lower speeds of walking in particular when the perturbation is applied in early stance thus maximizing the time for instability to develop. Wang and Srinivasan [22] have indicated that as much as 80% of the variance in deviations of foot placement from the average during unperturbed walking at speeds ranging from 1 to 1.4 m/s could be explained by deviations from the average in pelvis position and speed at midstance. Vlutters et al. [23] have also shown a similar correlation for perturbed walking at speed of 1.2 m/s. However, a study from Stimpson et al. [24] that examined step-by-step control of step width during unperturbed walking at speeds ranging from 0.2 to 1.2 m/s has shown that the strength of the relationship between the step width and pelvis mechanics is significantly reduced at lower speeds. These findings imply that utilization of adequate foot placement (stepping strategy) as the main strategy to maintain dynamic stability in the frontal plane depends substantially on walking speed. Therefore, during very slow walking, which is characteristic for clinical populations, other balancing mechanisms, primarily the inertial strategy in a form of hip strategy, which are considered to have a limited control ability to counteract perturbations applied in the frontal plane during walking at normally used speeds [2], should be employed earlier in the stance to impede the development of instability and thus decisively contribute to successful correction of an outward perturbation.

Previous studies have shown that mediolateral ankle strategy is employed following an outward push regardless of walking speed and intensity of perturbation [4, 5, 13] probably because it can, according to the inverted pendulum model, act fast against the developing instability by increasing GRF_ML_ in the direction opposite to the action of a perturbation. This is also what we observed in this study. No visually noticeable movement of the trunk or the arms was observed following perturbations in our study, which is consistent with observations from other studies using similar perturbation intensities and at walking speeds of 1.2 m/s [4] and 0.6 m/s [5]. However, the observed impulse-like increase of GRF_ML_ immediately after the perturbation commencement at low walking speed is not consistent with the inverted pendulum model [1, 12] and implies movement of other body segments. Balancing activity during the “in-stance” period following the commencement of outward perturbation seems to be similar to balancing while standing on one leg [12, 17] where visually notable counter-rotation of body segments resulting in an increase of GRF_ML_ that acts in the opposite direction of the COM_ML_ movement is readily used to maintain balance. Our recent study [18] and the study from Vlutters et al. [19] have shown pronounced activity of hip abductors of the stance leg following an outward perturbation which seems to be the cause of the observed impulse-like increase in GRF_ML_ constituting a hip strategy. Since this GRF_ML_ impulse was rather small, it has limited capacity to substantially move the relatively heavy trunk. Future studies should explore the neuromechanics of the “in-stance” balancing responses (consisting of ankle and hip strategies) following an outward perturbation at low walking speed in more details.

### 4.2. Shortening and Prolongation of the Stance Duration as a Balancing Strategy

It was shown that humans while walking at normal speeds and when subjected to larger perturbations of inward direction react more quickly by shortening their stance duration so that the next step which is also a corrective step is made earlier [4, 11]. Our results show that this is also the case for lower speeds.

However, when reacting to outward perturbations at lower speeds, the stance phase was in most cases substantially prolonged, thus prolonging utilization of the “in-stance” hip strategy resulting in an increase of GRF_ML_ in the first half of the stance that acted in the direction opposite to the movement of COM.

### 4.3. Braking of the Movement in the Plane of Progression as a Balancing Strategy

Both types of perturbation lead to temporary slowing down of progression in the sagittal plane, which was more pronounced for outward perturbations at lower speeds and stronger perturbations. Similar results were also observed in the studies of Hof et al. [4, 11, 13], however, at higher walking speeds. It seems that slowing down of the movement in the plane of progression following perturbation acting in the frontal plane is related to stiffening of the ankle [13] and also knee and hip joints [19] of the stance leg.

### 4.4. Widening of Steps as a General Strategy to Increase Stability when Faced with the Prospect of a Period of Perturbations

Several studies have identified a common precautionary strategy of widening steps when faced with prospective perturbations [14, 15]. The results of our study show that the subjects consistently adopted wider steps during perturbed walking sessions. In our opinion, this further stresses the importance of utilizing the hip balancing strategies in the first half of the stance at the lowest speed of walking as seen in this study, since in a real-life situation, the occurrence of perturbation cannot be expected in advance. Therefore, the narrower stepping that is normally exercised during walking would facilitate an even larger destabilizing effect of a perturbation applied in the frontal plane as compared to walking with adopted wider stepping.

### 4.5. Relevance of COM-Based Pushes to Real-Life Situations

Hof and Duysens [13] compared the results of ankle muscle activity responses obtained in their study, where the pushes at the level of COM were applied, to the ankle muscle activity obtained in studies that used walking surface translations as a source of perturbation. They concluded that the lateral translation of the floor is comparable to inward (medial) perturbation at the level of the waist while the medial translation of the floor is similar to outward (lateral) perturbation at the waist level. Oddsson et al. [8] applied surface translations and observed that lateral translation of the standing foot during the midstance caused the upcoming step to be wider while a medially directed translation caused the upcoming step to be narrower. This is also in agreement with the notion of Hof and Duysens [13] with respect to the similarity of surface translation-based and waist push-based perturbations.

### 4.6. Methodological Considerations and Study Limitations

We limited our analysis to single perturbation timing. The instant of perturbation commencement at the beginning of the stance was selected because this particular timing gives the perturbation opportunity to develop instability for the longest period until the leg in swing can enter stance onto a new location. For example, Oddsson et al. [8] have applied perturbations during the midstance and not at the beginning of the stance phase when one would normally expect a real slip to occur. They did that in order to avoid possible tripping which is clearly associated with the stepping response following an outward perturbation. Therefore, a perturbation applied at the beginning of the stance seems to be the most challenging to cope with. Additionally, at this perturbation timing, the responses throughout the varied speeds and intensities finished at the end of the next step (within one stride—0–100% of a gait cycle). This enabled consistent treatment of all responses. However, from a methodological point of view, the inclusion of two additional instances of perturbation occurrence (at 30% and 60% of stance) increased the level of unpredictability which increases the strength of our findings.

The largest perturbation intensity used in this study was 15% of body weight which was also the value used in our previous study [7]. At this perturbation intensity, no noticeable trunk movement or arm movement was observed; however, at the lowest treadmill speed, a perturbation of 15% elicited responses in some subjects that, beside hip strategy, also required utilization of stance foot repositioning through pivoting on the toes and heel which has also been shown in our previous study [14]. This response indicates that if we increased the perturbation magnitude even further, additional balancing strategies such as trunk rotation, rapid arm and leg movements, and possibly also hopping on the stance leg could be employed, possibly resulting in inconsistent and variable within-subject responses.

The balance assessment robot was controlled such that the interaction forces between the walking subject and pelvis link were as low as possible. We have assessed the peak interaction forces in a previous study and found that the influence of these forces on COP and GRF in sagittal and frontal planes as well as on EMGs of major lower limb muscles during unperturbed walking had negligible effects in the range of walking speed from 0.4 to 0.8 m/s [25]. In another study, we have demonstrated that the interaction between the balance assessment robot and the pelvis of a walking subject is purely passive; thus, the interaction forces can be perceived as reflected inertia which was estimated for the balance assessment robot to be below 5 kg at walking speed of 0.85 m/s [7]. This is below a value of 6 kg identified in the study of Meuleman et al. [26] that can be added to the pelvis without significantly affecting the gait. Therefore, we may conclude that the interaction forces between the balance assessment robot and the walking subject had minimal influence on the dynamic balancing responses observed in this study.

A valid question one can pose is how relevant may be the balancing responses assessed in healthy people to individuals with gait pathology. Able-bodied and neurologically impaired subjects have in general different repertoires of available muscle actions to react to unexpected mechanical perturbations. Balancing responses as assessed in able-bodied subjects can be regarded as optimal solution for a given speed of walking and a given perturbation strength. In this study, we found differences in a way how the able-bodied population reacts to perturbations of the same intensity at different speeds of walking. A significant balancing activity must commence already during the “in-stance period,” which is not the case for higher walking speeds. This implies that at low speed of walking, the task of balancing for neurologically impaired will be substantially the same as for able-bodied, and if they will not react appropriately already in the “in-stance period,” this will have consequences for subsequent phases of response. Therefore, understanding balancing responses in able-bodied individuals at speeds that are similar to those used by neurologically impaired individuals enables us to better understand what the task at hand is for neurologically impaired when subjected to mechanical perturbation.

## 5. Conclusion

In conclusion, our findings reveal reactive dynamic balancing responses following perturbations delivered at the waist at very slow walking speeds. The responses to inward perturbations predominantly consist of a stepping strategy at all walking speeds and all tested perturbation intensities. The responses to outward perturbations are highly dependent on the walking speed and perturbation intensity. Our interpretation is that at the slowest speed and lowest intensity, the hip strategy is dominant while at the greatest speed and highest intensity, the stepping strategy dominates. Between these two extremes, a synergy of both strategies is used with the relative share of each strategy depending on the walking speed and perturbation intensity. Further studies should explore in more details the neuromechanics of the “in-stance” balancing responses (consisting of ankle and hip strategies) following an outward perturbation at low walking speeds.

The results related to balancing responses following outward perturbations have implications for the development of a screening method which could identify potential fallers among either elderly or neurologically impaired populations. Inability to generate an adequate “in-stance” response at slower speeds of walking could be an indication of diminished balancing abilities. The results of this study may also be very relevant to the developers of control approaches applied in robot exoskeletons that are used to support walking and balance functions.

## Data Availability

The datasets used and/or analyzed during the current study are available from the corresponding author on request for reasonable use.
